# Prior Cytomegalovirus Infection Shapes Lymphocyte Activation and Function During Pregnancy

**DOI:** 10.3390/ijms27073257

**Published:** 2026-04-03

**Authors:** Miguel Ângelo-Dias, Catarina Gregório Martins, Mariana Apolinário Mata, Madalena Barata, Ana Chung, Susana Sarzedas, Élia Fernandes, Augusta Marques, Maria de Jesus Chasqueira, Paulo Paixão, Jorge Lima, Luis Miguel Borrego

**Affiliations:** 1 Immunology Department, NOVA Medical School, Faculdade de Ciências Médicas, NMS, FCM, Universidade NOVA de Lisboa, 1169-056 Lisbon, Portugal; miguel.dias@nms.unl.pt (M.Â.-D.);; 2 Comprehensive Health Research Centre—CHRC, NOVA Medical School, Faculdade de Ciências Médicas, NMS, FCM, Universidade NOVA de Lisboa, 1169-056 Lisbon, Portugal; 3 Department of Obstetrics and Gynecology, High-Risk Pregnancy Centre, Hospital da Luz Lisboa, 1500-650 Lisbon, Portugal; 4 Infection Department, NOVA Medical School, Faculdade de Ciências Médicas, NMS, FCM, Universidade NOVA de Lisboa, 1169-056 Lisbon, Portugal; 5 Department of Immunoallergy, Hospital da Luz Lisboa, 1500-650 Lisbon, Portugal

**Keywords:** cytomegalovirus, pregnancy, immune profile, T cells, B cells, cytokines

## Abstract

Pregnancy represents a dynamic immunological state in which the maternal immune system must balance tolerance toward the semi-allogeneic fetus while maintaining antimicrobial defense. Cytomegalovirus (CMV) infection is highly prevalent worldwide and profoundly shapes immune cell differentiation and long-term activation in adults. However, its interaction with pregnancy-associated immune remodeling remains incompletely defined. In this prospective longitudinal study, we comprehensively analyzed immune profiles of healthy pregnant women across all three trimesters and age-matched nonpregnant controls, stratified by CMV IgG serostatus. Multiparametric flow cytometry characterized T and B cell subsets and cytokine production following in vitro stimulation, while circulating cytokines and adhesion molecules were quantified using multiplex immunoassay. Gestational age was the primary determinant of leukocyte dynamics. Nevertheless, CMV-seropositive pregnant women showed enhanced activation and differentiation of CD4^+^ and, more prominently, CD8^+^ T cell subsets, changes not observed in nonpregnant women. Despite pronounced cellular differences, serum cytokine and adhesion molecule levels were largely comparable between CMV-seropositive and CMV-seronegative participants in both pregnant and nonpregnant groups. Functionally, CMV-seropositive women exhibited enrichment of IFN-γ– and IL-21–producing T cells, whereas B cell responses remained predominantly IL-10–dominated. These findings indicate selective alterations in maternal lymphocyte activation and function during pregnancy in CMV-seropositive women, without evidence of systemic inflammation.

## 1. Introduction

Pregnancy constitutes a distinct immunophysiological condition requiring the maternal immune system to ensure tolerance toward the fetus while preserving host protection against pathogens. Contrary to the long-held view of global immunosuppression, pregnancy is now recognized as a finely tuned immunoregulatory process characterized by stage-specific immune adaptations [1,2,3]. Early gestation is characterized by a mild pro-inflammatory milieu that supports implantation and placentation, followed by immune quiescence and tolerance during the mid-trimester to support fetal growth, while late pregnancy is associated with a reactivation of inflammatory pathways in preparation for parturition. Disruption of this immunological balance has been implicated in major obstetric complications such as miscarriage, preeclampsia, and preterm birth [4,5].

Human cytomegalovirus (CMV) is a widespread β-herpesvirus that establishes lifelong latency after primary infection [6]. CMV infection is typically asymptomatic in immunocompetent individuals and shifts to a latent state, which may reactivate sporadically later in life [7]. CMV latency is maintained through complex immune evasion strategies, including modulation of antigen presentation, cytokine signaling, and T cell activation pathways. Simultaneously, CMV infection is associated with chronic, low-grade immune activation and long-term expansion of highly differentiated T cell populations, a phenomenon often referred to as “memory inflation” (for review, see [8]). Collectively, these features make CMV serostatus a major determinant of immune phenotype in adult populations.

CMV is also the most common viral cause of congenital infection worldwide, affecting 0.2 to 2.2% of live births [9,10], and remains the leading non-genetic cause of sensorineural hearing loss [11]. Among women of reproductive age, IgG seroprevalence ranges from approximately 70% to over 90%, depending on geographic and socioeconomic factors [6]. While the consequences of primary CMV infection during pregnancy for the fetus are well documented, considerably less is known about the effects of prior CMV infection, defined by CMV IgG seropositivity, on maternal immune regulation throughout gestation. Importantly, pregnancy itself induces substantial changes in immune cell composition and function, raising the possibility that gestational immune modifications may have an impact on CMV-driven immune signatures.

In nonpregnant CMV-seropositive adults, expansions of polyfunctional and highly differentiated CD4^+^ and CD8^+^ T cell subsets, increased expression of activation and exhaustion markers, and coordinated changes in natural killer (NK) cell populations have been consistently observed, reflecting the long-term immunological imprint of CMV [12,13,14]. In contrast, alterations within the B cell compartment appear more limited and context dependent [15,16,17]. During pregnancy, available data suggest that CMV serostatus may influence maternal T cell differentiation and activation profiles, particularly during late pregnancy [18], while different anti-CMV serological patterns have distinct characteristic patterns of CD8 T cell phenotypes and antigen recognition during pregnancy [19]. However, longitudinal studies capturing immune trajectories across gestation remain scarce, and functional characterization of lymphocyte responses in this context is particularly limited.

Understanding the interface between CMV-driven and pregnancy-driven immune programs is essential for several reasons. First, CMV serostatus may represent a significant confounding factor when interpreting immune phenotypes in pregnant cohorts. Second, CMV-associated immune activation could influence maternal responses to other infections or immunological challenges during gestation. Finally, defining the extent to which pregnancy interacts with the established CMV-driven immune signatures may provide insight into inter-individual variability in maternal immune adaptation and pregnancy outcomes.

In the present study, we performed a comprehensive longitudinal analysis of T and B cell immune profiles and functions in healthy pregnant women across all trimesters of gestation, stratified by CMV IgG serostatus, and compared these profiles with age-matched nonpregnant controls. Combining multiparametric immunophenotyping, serum cytokine profiling, and cell polyfunctionality analyses, we aimed to delineate how CMV serostatus intersects with pregnancy-induced immune remodeling to shape maternal immune landscapes over gestation.

## 2. Results

### 2.1. Study Population

A total of 64 pregnant women and 30 nonpregnant women were initially recruited. Among pregnant participants, 11 women were excluded from the final analysis because of pregnancy complications (*n* = 5) or nonclinical loss to follow-up (*n* = 6), resulting in a final cohort of 53 pregnant women. Of the expected longitudinal samples, seven were not collected due to nonclinical reasons. All nonpregnant women completed the study visit. CMV serostatus was determined by the presence of anti-CMV IgG and IgM antibodies. Among pregnant women, 43 (81.1%) were CMV-seropositive, and 10 (18.9%) were CMV-seronegative. In the nonpregnant group, 25 women (83.3%) were CMV-seropositive, and 5 (16.7%) were CMV-seronegative. Anti-CMV IgM was undetectable among all participants, and no seroconversion occurred during the study period. In addition, CMV DNA was not detected in plasma samples, confirming the absence of overt systemic viral reactivation.

Demographic and clinical characteristics stratified by CMV serostatus are summarized in Table 1. No significant differences were observed between groups with respect to age, BMI, blood pressure, parity, or miscarriage history, mode of delivery, neonatal birth weight, or fetal sex. Gestational age at delivery was slightly shorter in CMV-seropositive pregnant women compared with CMV-seronegative women. Apgar scores at 1 min were lower in CMV-seronegative pregnancies, although 5 min scores were comparable across groups and within the normal clinical range.

Anti-CMV IgG concentrations remained stable across pregnancy trimesters in CMV-seropositive women and did not differ longitudinally. However, IgG levels were significantly lower in pregnant compared with nonpregnant CMV-seropositive women.

### 2.2. Leukocyte and Platelet Dynamics Across Pregnancy by CMV Serostatus

**Figure 1:**
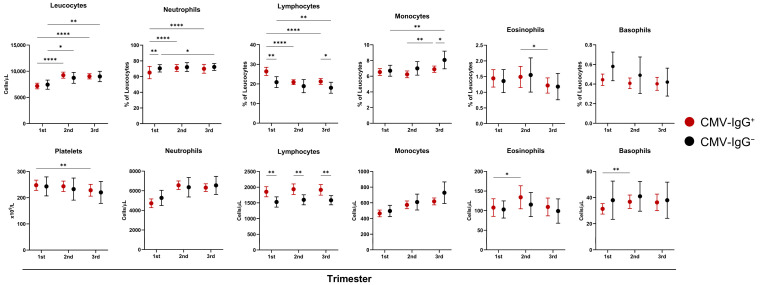
Leukocyte and platelet variations during pregnancy according to CMV serostatus. Total leukocyte, platelet, and leukocyte subset counts (bottom panels) and relative frequencies (top panels) in CMV-seropositive (CMV-IgG^+^, red) and CMV-seronegative (CMV-IgG^−^, black) pregnant women across the three trimesters of gestation. Data are shown as mean ± 95% CI. Statistical significance was determined with mixed-effects analysis followed by Tukey’s multiple comparisons test, and is indicated as: * *p* < 0.05, ** *p* < 0.01, **** *p* < 0.0001. Detailed information and additional comparisons are provided in Supplementary Tables S1 and S2.

Total leukocyte and platelet counts, as well as leukocyte subset distributions, were analyzed longitudinally across pregnancy by CMV serostatus (Figure 1; Supplementary Tables S1 and S2).

Gestational age exerted a strong effect on overall leukocyte composition. Total leukocyte counts increased progressively from the first to the third trimester, reflecting physiological pregnancy-associated leukocytosis. This increase was primarily driven by rising neutrophil counts and proportions, accompanied by a gradual decline in lymphocyte percentages across gestation. Monocytes and eosinophil proportions showed more modest increases during later trimesters, whereas basophil frequencies remained largely unchanged. Platelet counts decreased slightly across pregnancy, consistent with known gestational trends.

CMV serostatus modestly modulated these trajectories. CMV-seropositive pregnant women exhibited higher absolute lymphocyte counts across all trimesters compared with CMV-seronegative women, while relative lymphocyte frequencies were also increased. Conversely, CMV-seropositive women displayed lower neutrophil proportions during early pregnancy and increased monocyte proportions during late pregnancy. Eosinophil and basophil populations and platelet counts did not differ significantly between CMV-seropositive and CMV-seronegative participants.

Overall, the gestational stage represented the dominant determinant of leukocyte dynamics. CMV seropositivity was associated with subtle but consistent modulation of lymphocyte and monocyte compartments throughout pregnancy.

### 2.3. CMV Serostatus Is Associated with Altered Lymphocyte Activation and Differentiation During Pregnancy

**Figure 2:**
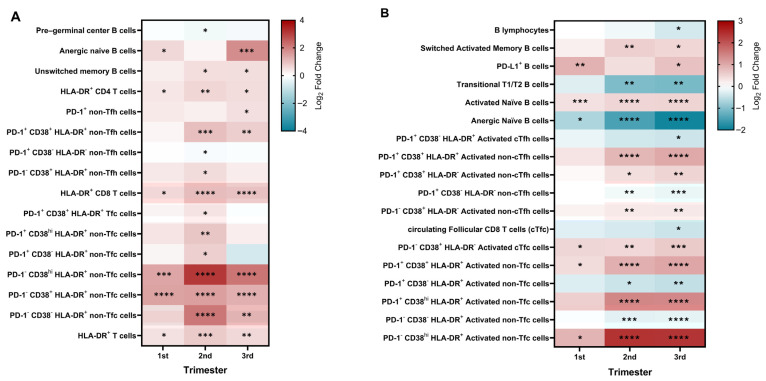
CMV-associated immune profile modifications across pregnancy. Heatmaps show differences in the relative frequencies of selected immune cell subsets across the three trimesters. Panel (**A**) compares CMV seropositive (CMV-IgG^+^) and seronegative (CMV-IgG^−^) pregnant women, while panel (**B**) compares CMV-IgG^+^ pregnant and CMV-IgG^+^ nonpregnant women. The color gradient represents the mean log_2_ fold change (CMV-IgG^+^ pregnant women relative to CMV-IgG^−^ pregnant women in (**A**), or relative to CMV-IgG^+^ nonpregnant women in (**B**), with red indicating higher and blue indicating lower values in CMV-IgG^+^ pregnant women. Statistical significance was assessed using an unpaired *t*-test with Welch’s correction (**A**) and one-way ANOVA with Dunnett’s multiple comparisons test (**B**) and is indicated as follows: * *p* < 0.05, ** *p* < 0.01, *** *p* < 0.001, **** *p* < 0.0001. Detailed longitudinal comparisons across pregnancy stages are provided in Supplementary Tables S1 and S2.

To investigate whether CMV serostatus influenced adaptive immune cell composition beyond global leukocyte changes, we analyzed the frequencies and absolute counts of selected T and B cell subsets across pregnancy (Figure 2; Supplementary Tables S1 and S2).

Within the B cell compartment, CMV-seropositive pregnant women exhibited higher absolute counts of both unswitched and switched memory B cells, most prominently during the second trimester. Unswitched memory B cell frequencies were also modestly increased during the third trimester. In addition, increased proportions of anergic naïve B cells (CD21^−^CD24^+^CD38^+^) were observed in CMV-seropositive women during the first and third trimesters (Figure 2A).

More pronounced CMV-associated differences were observed within the T cell compartment. CMV-seropositive pregnancy, compared to CMV-seronegative pregnancy, was characterized by increased frequencies and absolute counts of activated CD4^+^ and CD8^+^ T cell subsets expressing HLA-DR, PD-1, and CD38 (Figure 2A). These differences were particularly evident among non-follicular T cells, including PD-1^+^CD38^+^HLA-DR^+^ non-Tfh and PD-1^−^CD38^−/+/hi^HLA-DR^+^ non-Tfc subsets, and became more marked toward later gestation. Differences observed in absolute counts may reflect, at least in part, the lower total lymphocyte numbers detected in CMV-seronegative women, rather than selective depletion of specific subsets (Supplementary Table S2).

Collectively, these data indicate that CMV-seropositive women exhibit sustained modulation of lymphocyte activation and differentiation trajectories throughout pregnancy, with several CMV-related alterations becoming more evident during late gestation.

To determine whether CMV-associated immune alterations were specific to pregnancy, lymphocyte subsets were compared between CMV-seropositive and CMV-seronegative nonpregnant women. Except for total CD8^+^ T cell frequencies, relatively few differences in lymphocyte subsets were observed between these groups (Supplementary Table S1). Owing to the limited number of CMV-seronegative nonpregnant participants, this comparison was considered exploratory but is consistent with the notion that most CMV-associated immune alterations identified in this study become apparent primarily in the context of pregnancy rather than reflecting baseline differences present in the nonpregnant state.

To further dissect the relative contributions of viral serostatus and pregnancy, CMV-seropositive pregnant women were directly compared with CMV-seropositive nonpregnant women (Figure 2B, Supplementary Tables S1 and S2). Significant differences were observed across most of the analyzed immune parameters, indicating that pregnancy profoundly modifies CMV-associated immune phenotypes. These differences were most pronounced within the T cell compartment and were particularly evident among CD8^+^ T cell subsets expressing markers of activation and differentiation, including HLA-DR, CD38, and PD-1.

Notably, the differences between pregnant and nonpregnant women were substantially more extensive within the CMV-seropositive group than within the CMV-seronegative group. Whereas comparison of CMV-seronegative pregnant and nonpregnant women revealed relatively few alterations in lymphocyte parameters, CMV-seropositive pregnant women differed from their nonpregnant counterparts across the majority of analyzed immune variables, particularly within the CD8^+^ T cell compartment.

Longitudinal comparisons across pregnancy were also performed for all immune parameters and are detailed in Supplementary Tables S1 and S2. Temporal changes across trimesters were more evident among CMV-seropositive participants, whereas fewer statistically significant longitudinal differences were observed in CMV-seronegative women.

Together, these findings suggest that the immune modulation observed arises from the combined influence of CMV serostatus and pregnancy, rather than from either factor alone, with the most pronounced combined effects observed in the CD8^+^ T cell compartment.

### 2.4. Dynamics of Serum Cytokines Throughout Pregnancy in CMV Seropositive and Seronegative Women

**Figure 3:**
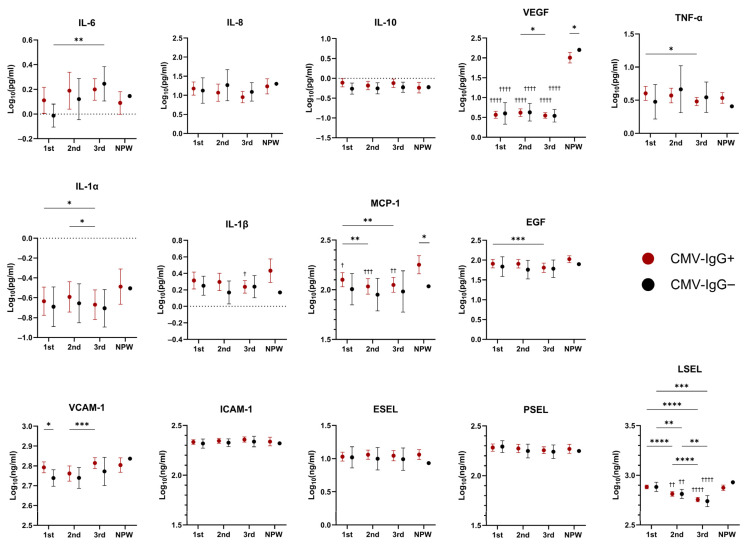
Variations in soluble factors during pregnancy according to CMV serostatus. Serum concentration of cytokines (IL-6, IL-8, IL-10, TNF-α, IL-1α, and IL-1β), chemokine (MCP-1), growth factors (VEGF, EGF), and adhesion molecules (VCAM-1, ICAM-1, ESEL, PSEL, LSEL) in CMV-seropositive (CMV-IgG^+^, red) and CMV-seronegative (CMV-IgG^−^, black) women across pregnancy trimesters and in nonpregnant women (NPW). Data are presented as mean with 95% confidence interval in log_10_(pg/mL) or log_10_(ng/mL). Significant differences within and between each group across time points were analyzed using a mixed-effects model with the Geisser-Greenhouse correction, followed by Tukey’s multiple comparisons test, and are indicated by asterisks (* *p* < 0.05, ** *p* < 0.01, *** *p* < 0.001, **** *p* < 0.0001). Comparisons between pregnant time points and the corresponding CMV-IgG^+^ or CMV-IgG^−^ nonpregnant group were performed using ordinary one-way ANOVA followed by Dunnett’s multiple comparisons test and are indicated by crosses (^†^ *p* < 0.05, ^††^ *p* < 0.01, ^†††^ *p* < 0.001, ^††††^ *p* < 0.0001).

To determine whether CMV-associated cellular differences were accompanied by alterations in systemic immune mediators, serum concentrations of cytokines, chemokines, growth factors, and adhesion molecules were quantified longitudinally (Figure 3).

Across pregnancy, serum profiles appeared to be dominated by gestational effects rather than CMV serostatus. Indeed, except for increased vascular cell adhesion molecule-1 (VCAM-1) concentrations during the first trimester in CMV-seropositive women, no significant differences were detected between CMV-seropositive and CMV-seronegative pregnant women at individual time points. Trends toward higher IL-6 concentrations during early pregnancy in CMV-seropositive women did not reach statistical significance.

Longitudinal analyses revealed pregnancy-associated modulation of several mediators. Monocyte chemoattractant protein (MCP)-1 and epidermal growth factor (EGF) concentrations decreased progressively across gestation, and VCAM-1 levels increased during the third trimester in CMV-seropositive women, whereas interleukin (IL)-6 levels increased over time in CMV-seronegative women. Vascular endothelial growth factor (VEGF) and L-selectin (L-SEL) concentrations were markedly reduced during pregnancy in both CMV-seropositive and CMV-seronegative participants compared with nonpregnant controls, and declined further during later trimesters. Intercellular adhesion molecule (ICAM)-1, E-SEL, and P-SEL levels remained relatively stable across pregnancy regardless of CMV serostatus. Proinflammatory cytokines such as IL-1α, tumor necrosis factor (TNF)-α, and IL-1β showed minor fluctuations, while IL-10 remained unchanged throughout pregnancy. The cytokines IL-2, IL-4, and interferon (IFN)-γ showed high proportions of values below the lower limit of quantification (LLOQ) and were excluded from the analysis (Supplementary Table S3).

Overall, these findings suggest that CMV-seropositive women do not exhibit broad systemic inflammatory changes during pregnancy and that observed serum mediator dynamics primarily reflect physiological gestational adaptations.

### 2.5. Influence of CMV Serostatus and Pregnancy Progression on T Cell Cytokine Responses

**Figure 4:**
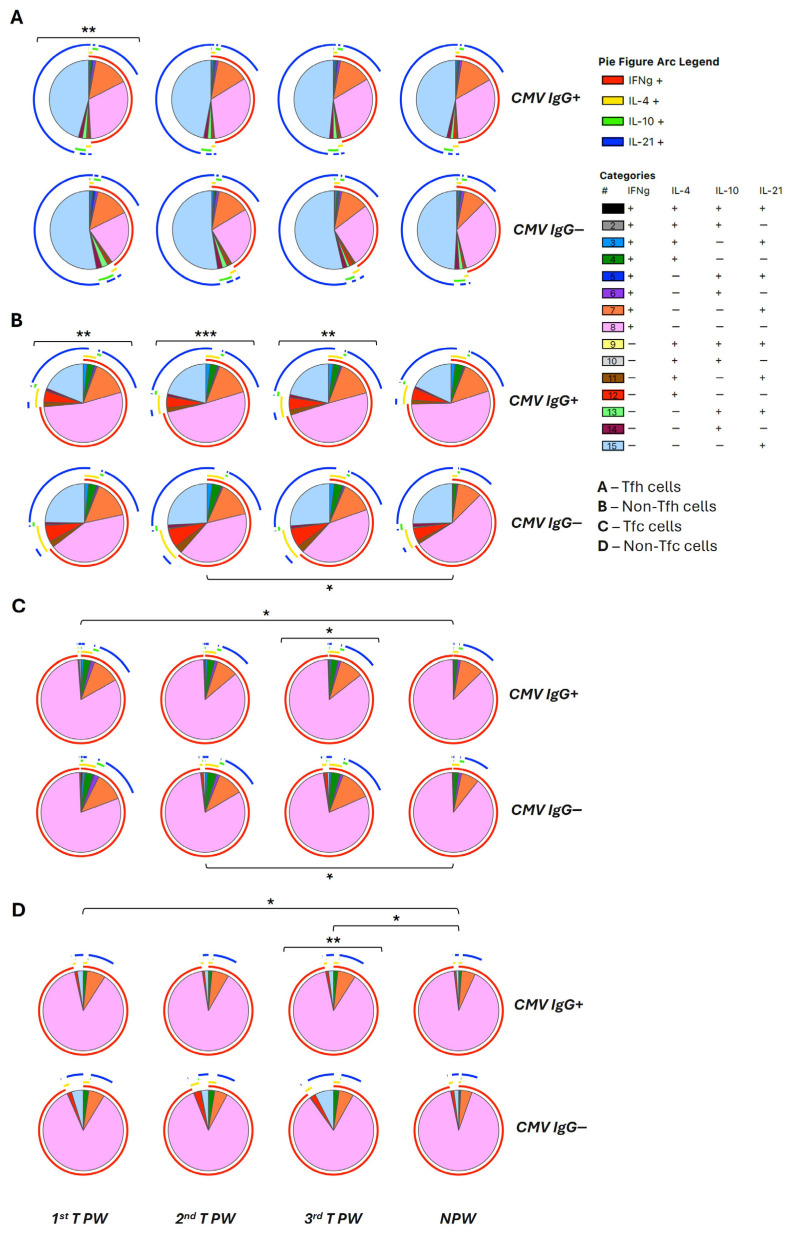
Polyfunctionality of CD4 and CD8 T cells after in vitro stimulation. Pie charts show the proportion of Tfh (**A**), non-Tfh (**B**), Tfc (**C**), and non-Tfc (**D**) cells producing different combinations of IFN-γ, IL-4, IL-10, and IL-21 following in vitro stimulation with PMA, LPS, and ionomycin. Data are presented according to pregnancy stage (1st, 2nd, and 3rd trimester pregnant women, and nonpregnant women) and CMV serostatus (CMV-IgG^+^ and CMV-IgG^−^). Values shown in the pie charts represent mean frequencies. All frequencies were background-corrected by subtracting values obtained in the non-stimulated condition. Statistical differences between pie charts (indicated by brackets) were assessed using the SPICE permutation test (* *p* < 0.05, ** *p* < 0.01, *** *p* < 0.001). PW, pregnant women; NPW, nonpregnant women.

To assess whether CMV serostatus affected lymphocyte functional capacity across gestation, intracellular cytokine production was analyzed following in vitro stimulation, and polyfunctionality patterns were evaluated using SPICE analysis (Figure 4; Supplementary Table S4).

Within the CD4^+^ T cell compartment, Tfh cells were predominantly characterized by IL-21^+^ and IFN-γ^+^ single-producing subsets (Figure 4A). During the first trimester, cytokine-expression profiles differed significantly between CMV-seropositive and CMV-seronegative pregnant women, driven primarily by differences in these two subsets (Supplementary Table S4). Non-Tfh cells exhibited broader cytokine-expression patterns and showed CMV-dependent shifts across all three trimesters (Figure 4B), with variations in IL-4^+^, IL-21^+^, and IL-4^+^IL-21^+^ producing cells (Supplementary Table S4).

Among CD8^+^ T cells, Tfc and non-Tfc subsets represented the principal sources of IFN-γ^+^ and IFN-γ^+^IL-21^+^-producing subsets. In Tfc cells, trimester-dependent differences were observed between pregnant and nonpregnant women in both CMV-seropositive and CMV-seronegative groups (Figure 4C), whereas CMV-associated differences were most evident within non-Tfc subsets during the third trimester, driven largely by increased proportions of IL-21^+^ single-producing cells (Figure 4D, Supplementary Table S4).

Highly polyfunctional T cells expressing three or more cytokines were infrequent across all groups. Nevertheless, statistically significant differences in overall cytokine-expression distributions were detected between CMV-seropositive and CMV-seronegative women at multiple pregnancy stages, indicating qualitative shifts in the functional potential, rather than expansion of rare highly polyfunctional populations.

B cell cytokine responses were dominated by IL-10 production, accounting for 1% of total B cells, whereas polyfunctional B cells were rare (Supplementary Figure S1). In CMV-seropositive women, the relative contribution of IL-10–producing B cells was consistently high, particularly during the first trimester and in nonpregnant women. Main differences were observed between early and late gestation, as well as between mid- and late gestation, compared with nonpregnant women, with IL-10^+^ and IFN-γ^+^ producing subsets accounting for most of these differences (Supplementary Table S4). Direct comparisons between CMV-seropositive and CMV-seronegative women revealed significant differences in overall cytokine-expression composition, particularly during later gestation and in the nonpregnant group.

Collectively, these functional analyses demonstrate that CMV serostatus is associated with pregnancy-stage-dependent modulation of T and B cell cytokine-production capacity, characterized predominantly by skewing toward IFN-γ and IL-21-producing T cell subsets.

## 3. Discussion

Cytomegalovirus infection is highly prevalent in adult populations and is well recognized for its capacity to remodel the adaptive immune system through long-term effects of lymphocyte differentiation, activation and function [20]. This capacity is relevant during pregnancy, as the consequences of primary infection for the fetus are well documented [21]. In the present longitudinal study, we demonstrate that CMV seropositivity interacts with pregnancy-induced immune remodeling to shape distinct maternal immune phenotypes across gestation. Importantly, our findings indicate that CMV serostatus and pregnancy do not act independently but instead exert combined effects on cellular immune composition and functional potential.

The most consistent CMV-associated differences observed in this study involved the T cell compartment, particularly CD8^+^ non-follicular subsets expressing activation, maturation, and differentiation markers. CMV-seropositive pregnant women exhibited increased frequencies and absolute counts of activated CD8^+^ T cells across gestation, with differences becoming more pronounced during later trimesters. Notably, these alterations were not evident when comparing CMV-seropositive and CMV-seronegative nonpregnant women, indicating that pregnancy modifies canonical CMV-driven immune signatures rather than merely unmasking baseline differences present outside gestation. Sustained elevations in progesterone and estrogens during gestation are known to influence T cell activation thresholds and differentiation pathways [22,23] and may therefore amplify or reshape pre-existing CMV-imprinted T cell populations. Thus, rather than acting independently, pregnancy and CMV serostatus may converge on overlapping pathways of T cell differentiation, generating a composite immune phenotype that is specific to CMV-seropositive gestation.

Although studies evaluating the impact of CMV serostatus during pregnancy remain limited, our results are consistent with observations by Lissauer et al. [18], who reported significant alterations in the maternal CD8^+^ T cell repertoire during pregnancy based on CCR7, CD45RA, and CD38 expression. In that study, CMV infection was associated with the accumulation of highly differentiated CMV-specific memory CD8^+^ T cells and with altered CD8^+^ T cell dynamics across gestation.

More recently, a study using MHC-dextramer–based single-cell RNA sequencing combined with flow cytometry further characterized CMV-specific CD8^+^ T cells in pregnant women with different serological profiles [19]. CMV-specific CD8^+^ T cells from CMV-IgG^+^IgM^−^ women predominantly exhibited effector memory and terminally differentiated effector memory (TEMRA) phenotypes, expressing cytotoxic molecules such as granzymes and perforin. This phenotype is consistent with long-term immunological surveillance and control of latent infection, in contrast to recent primary infection (IgG^+^IgM^+^), which was characterized by a predominance of effector T cells.

Although pregnancy-specific immune adaptations were not the primary focus of these studies, their findings, together with ours, underscore the importance of cellular immune profiling to complement serological assessments for improved risk stratification of congenital CMV.

In nonpregnant adults, latent CMV infection is known to drive the expansion of highly differentiated effector memory and terminally differentiated CD8^+^ T cell populations, a phenomenon often referred to as memory inflation [20,24]. We and others have previously reported increases in T cell activation and maturation profiles during late pregnancy compared to nonpregnant individuals [25,26,27,28,29], which in fact may, at least partially, reflect CMV serostatus influence rather than pregnancy-related immune adaptations alone, given the high prevalence of CMV seropositivity in the population. In this study, our findings are consistent with this established paradigm but further suggest that pregnancy-associated immune cues influence the magnitude and composition of these CMV-associated T cell populations, as reflected by differences observed between CMV-seropositive pregnant women and both CMV-seronegative pregnant women and CMV-seropositive nonpregnant women. Although we were unable to directly assess CMV-specific T cells, the observed phenotypic patterns align with previous reports describing CMV-driven accumulation of activated and differentiated CD8^+^ T cells during pregnancy [18,19]. Together, these data support the notion that CMV serostatus represents an important confounding variable when interpreting T cell phenotypes in pregnant cohorts.

CMV seropositivity has also been variably associated, albeit with conflicting results, with adverse pregnancy outcomes such as preeclampsia, preterm birth, and miscarriage [30,31,32,33]. If CMV-associated immune remodeling alters the inflammatory threshold during gestation, it may contribute to susceptibility or resilience to pregnancy complications. While CMV-driven enrichment of IFN-γ–producing T cells may enhance antiviral responsiveness and contribute to effective immune surveillance, excessive effector polarization could theoretically influence maternal–fetal immune tolerance, particularly in individuals predisposed to inflammatory pregnancy complications. Understanding whether and how CMV-induced immunological alterations during pregnancy influence maternal health, pregnancy outcomes, and responses to new infections is, therefore, of critical importance and warrants further investigation.

Interestingly, and in agreement with previous reports [18], despite pronounced CMV-associated differences in cellular immune composition, circulating concentrations of cytokines, chemokines, and soluble adhesion molecules were very similar between CMV-seropositive and CMV-seronegative pregnant and nonpregnant women. This dissociation between cellular activation and systemic mediator levels suggests that changes in immune cell differentiation and functional potential predominate rather than inducing overt systemic inflammation in CMV-seropositive individuals. Longitudinal changes observed in mediators such as IL-6, MCP-1, and TNF-α were consistent with physiological pregnancy-associated immune and vascular adaptations [34,35] and were largely independent of CMV serostatus.

VEGF dynamics during pregnancy remain controversial in the literature. While some studies report lower circulating VEGF concentrations during pregnancy, others describe elevated levels compared with nonpregnant women [34,36]. In the present study, VEGF concentrations were markedly reduced during pregnancy compared with the nonpregnant state in both CMV-seropositive and CMV-seronegative groups. This finding is consistent with reports describing reduced circulating VEGF during gestation and likely reflects increased production of soluble VEGF receptor-1 (sFlt-1), which sequesters free VEGF and limits its detectability in maternal serum. Given that VEGF exerts its primary effects locally within placental tissue to support angiogenesis and placental development, reduced circulating levels are considered a physiological feature of normal pregnancy rather than an indicator of impaired vascular function [37].

Functional analyses further demonstrated qualitative differences in cytokine-production capacity following in vitro stimulation according to CMV serostatus. Across CD4^+^ and CD8^+^ T cell subsets, CMV-seropositive pregnancy was characterized by relative enrichment of IFN-γ– and IL-21–producing cells. Highly polyfunctional T cells expressing three or more cytokines were infrequent in all groups. However, significant differences in overall cytokine-expression patterns were detected across pregnancy stages and according to CMV serostatus. CMV-seropositive women consistently exhibited skewing toward IFN-γ–producing CD4^+^ and CD8^+^ T cell subsets, suggesting enhanced antiviral capacity, alongside increased IL-21 production within follicular helper compartments. The increased IL-21 production observed within follicular helper compartments may provide mechanistic support for the expansion of memory B cell subsets detected during the second trimester, given the established role of IL-21 in B cell differentiation and antibody maturation. These findings extend previous observations in nonpregnant adults by demonstrating that CMV-associated functional skewing is modulated across gestation.

To our knowledge, this study represents the first analysis of CMV-associated T cell polyfunctionality in the context of pregnancy. Our findings are consistent with prior reports describing high IFN-γ expression by CD4^+^ and CD8^+^ T cells in chronic CMV infection among nonpregnant individuals [13,38,39]. Although the relatively small cohort size may have limited our ability to detect subtle pregnancy-related changes, most observed differences in cytokine-expressing subsets occurred between CMV-seropositive and CMV-seronegative groups, supporting a dominant role of CMV serostatus in shaping cytokine production capacity.

B cell cytokine responses were dominated by IL-10 production, with limited polyfunctionality; however, CMV-seropositive women displayed detectable differences in cytokine-expression patterns, reinforcing the concept of altered humoral immune dynamics.

Although alterations within the B cell compartment were more modest than those observed for T cells, CMV-seropositive pregnant women exhibited increased unswitched and switched memory B cell populations, particularly during the second trimester. Whether this more differentiated profile enhances antibody responses during gestation remains to be determined. These findings contrast with those of Dauby et al. [17], who reported expansions of CD27^+^CD20^+^CD21^low^ and CD27^−^CD20^+^CD21^low^ B cell subsets during primary CMV infection but observed minimal changes during CMV latent infection in nonpregnant individuals. Similarly, Goldeck et al. [16] reported minimal differences in the frequencies of peripheral B cell phenotypes associated with CMV seropositivity in both younger and older adults. Methodological differences and the dynamic immunological environment of pregnancy may account for some discrepancies.

Several limitations of this study should be acknowledged. First, the relatively small number of CMV-seronegative participants limits statistical power and generalizability, particularly for subgroup analyses. However, given the high global seroprevalence of CMV, recruitment of seronegative individuals remains inherently challenging. Larger and more balanced cohorts will be required to validate our observations. Second, the absence of CMV-specific phenotypic and functional assays precludes direct attribution of observed immune alterations to virus-specific lymphocytes. Incorporation of CMV-specific functional assays and additional markers (e.g., CD57) would provide deeper insight. Nevertheless, the confirmation of CMV seropositivity in the absence of detectable viremia, together with the inclusion of CMV-seropositive matched nonpregnant controls, strengthens the interpretation that pregnancy modifies CMV-associated immune signatures. Importantly, CMV DNA was not detected in plasma samples from seropositive participants by PCR at any time point, arguing against overt systemic viral reactivation during the study period. However, CMV DNA was not assessed in other biological compartments, such as urine or cervical secretions, and therefore localized and intermittent viral reactivation within tissue compartments cannot be excluded. In fact, previous studies suggest that CMV persistence may involve episodic viral gene expression or localized reactivation events that occur without systemic viremia but are nevertheless sufficient to sustain immune stimulation. These processes are thought to contribute to the maintenance of highly differentiated CMV-specific T-cell populations and to long-term immune modulation in seropositive individuals [7,24]. During pregnancy, such localized reactivation events could potentially contribute to the activation or reshaping of maternal immune responses, including the stimulation of tissue-resident or circulating immune cells, thereby influencing the immunological adaptations that characterize gestation. These processes represent important features that could be further explored in future studies incorporating tissue-level and functional immune assessments. Third, placental characteristics (i.e., weight and histopathological features) were not evaluated in this study. Placental assessment can provide important insights into the impact of CMV infection on placental structure and function, as CMV has been associated with histopathological alterations such as villitis, chronic inflammation, and vascular changes [40,41]. However, in the present cohort, pregnancies were clinically uncomplicated, and no obstetric or fetal indications arose that would warrant routine placental examination. Nevertheless, future studies incorporating systematic placental assessment may provide additional insight into the potential effects of CMV infection on placental function and its contribution to maternal immune modulation during pregnancy. Finally, other latent viruses, such as Epstein–Barr virus, were not assessed and may contribute to interindividual variability in immune phenotypes. Future studies incorporating multi-virus profiling would help clarify the interplay between chronic viral infections and gestational immunity.

Given the high global seroprevalence of CMV and its substantial impact on T cell differentiation, failure to account for CMV serostatus may confound the interpretation of immune phenotypes in pregnant cohorts. Our findings support the notion that part of what has historically been attributed to pregnancy-induced immune activation may, in fact, reflect underlying chronic viral imprinting. Future studies investigating immune adaptations during pregnancy should therefore consider systematic stratification by CMV serostatus to disentangle pregnancy-specific immune remodeling from CMV-associated immune effects. Still, the clinical implications of CMV-associated immune modulation during pregnancy remain to be fully elucidated. While CMV-driven immune activation could enhance antiviral responsiveness, it may also influence maternal–fetal immune tolerance. Future studies incorporating larger cohorts, virus-specific functional analyses, and clinical outcome measures will be essential to determine whether CMV seropositivity confers protective, neutral, or potentially adverse effects during pregnancy.

## 4. Materials and Methods

### 4.1. Study Design and Participants

This prospective observational study enrolled healthy pregnant women during the first trimester of pregnancy and followed them longitudinally until delivery. Participants were recruited at Hospital da Luz Lisboa between June 2022 and April 2025. Eligible women were aged 18 to 45 years, had naturally conceived singleton pregnancies, and completed at least two study visits without clinically related withdrawal. Gestational age at enrolment was determined by first-trimester ultrasound or by the date of the last menstrual period. An age-matched control group of healthy nonpregnant women was sequentially recruited during routine annual gynecological consultations and sampled once.

Exclusion criteria for both groups included chronic diseases (diabetes mellitus, hypertension, autoimmune disorders or malignancy), ongoing infection (hepatitis viruses, HIV, or CMV reactivation), use of immunomodulatory medication, or smoking within six months prior to blood sampling. Pregnant women were further excluded if they had a history of or presented with obstetric complications (preterm birth, fetal growth restriction, preeclampsia, neonatal ventilation, or recurrent pregnancy loss) or if they used medication other than prenatal supplementation (vitamins, folic acid, or iron).

All participants provided written informed consent prior to inclusion. Ethical approval was obtained from the Ethics Committees of Hospital da Luz Lisboa (CES/49/2021/ME) and NOVA Medical School (167/2021/CEFCM). The study was conducted in accordance with the Declaration of Helsinki [42]. All clinical and laboratory data were anonymized prior to analysis.

### 4.2. Study Visits

Pregnant participants attended three study visits corresponding to routine clinical assessments during the first (9th–12th weeks of gestation), second (24th–28th weeks of gestation, and third (after 32nd week of gestation) trimesters. Nonpregnant women attended a single study visit. At each visit, peripheral venous blood was collected into EDTA-coated, heparinized, and anticoagulant-free tubes.

Baseline demographic data (age), anthropometric measurements (body mass index, BMI), and clinical parameters (obstetric history, systolic and diastolic blood pressure) were recorded at enrollment. Additional clinical data, including complete blood counts and CMV IgG and IgM serostatus, were obtained from hospital medical records at each visit.

For pregnant women, delivery-related outcomes were recorded, including gestational age at delivery and mode of delivery. Neonatal data included sex, birth weight, and Apgar scores at 1 and 5 min. Apgar scores were interpreted according to standard clinical criteria, with values between 7 and 10 indicating normal neonatal adaptation.

### 4.3. Immunophenotyping

Peripheral blood immunophenotyping was performed using two pre-validated eight-color monoclonal antibody panels (14 distinct markers in total) designed for the identification and quantification of T and B cell subsets. Both panels were provided as lyophilized single-tube antibody mixes from ExBio (Praha, Czech Republic) and processed according to the manufacturer’s instructions. Briefly, 100 μL of whole blood was pre-washed with BD FACS Flow (BD Biosciences, San Jose, CA, USA), incubated with antibody mixtures for 20 min at room temperature, and subsequently subjected to erythrocyte lysis with EXCELLYSE Easy solution for 10 min at room temperature. Cells were washed with BD FACS Flow for 5 min at 300× *g* and analyzed on the flow cytometer within 30 min of staining completion.

A total of 74 lymphocyte subsets were quantified and expressed as absolute counts and/or proportions of their respective parent populations. B cell subsets were defined according to the classification framework proposed by Sanz and colleagues [43]. Comprehensive details of antibody panels and subset definitions are provided in Supplementary Tables S5 and S6. Representative gating strategies are shown in Supplementary Figures S2–S5.

### 4.4. Serum Cytokines and Soluble Mediators

Serum was separated from anticoagulant-free tubes by centrifugation at 900× *g* for 10 min, aliquoted, and stored at −80 °C until analysis. Serum samples were analyzed for a panel of cytokines, chemokines, growth factors, and adhesion molecules using the Multiplex Biochip Array Technology (Randox Laboratories Ltd., Crumlin, UK). The high-sensitivity cytokine and growth factors array included interleukin (IL)-2, IL-4, IL-6, IL-8, IL-10, IL-1α, IL-1β, TNF-α, IFN-γ, MCP-1, VEGF, and EGF. In parallel, soluble adhesion molecules, including VCAM-1, ICAM-1, E-selectin, P-selectin, and L-selectin, were quantified using the corresponding Randox adhesion molecule array.

All assays were performed according to the manufacturer’s instructions, and biochips were read using an Evidence Investigator™ analyzer (Randox Laboratories Ltd., Crumlin, UK). Serum samples were analyzed in single technical replicates, with all samples from the same participant assayed within the same analytical run to minimize inter-assay variability. To ensure assay performance and reproducibility, quality control samples (Randox Cytokine Multianalyte Controls, levels I, II, and III) were included in all runs. Measured values for all quality control samples were consistently within the acceptable ranges specified by the manufacturer. For patient samples with concentrations exceeding the upper limit of quantification (ULOQ), values were set to the ULOQ for statistical analysis purposes. Samples with concentrations below the limit of detection (LOD) were considered non-detectable and excluded from quantitative analyses. Soluble factors with more than 50% of values below the LLOQ were not considered for quantitative analysis between groups.

### 4.5. Intracellular Cytokine Staining and Functional Assays

To assess cytokine production capacity, intracellular cytokine staining was performed on heparinized whole-blood samples. Samples were diluted 1:1 with Iscove’s Modified Dulbecco’s Medium (IMDM, Corning^®^, Corning, NY, USA) and incubated for 5 h at 37 °C in a humidified 5% CO_2_ atmosphere, either unstimulated or stimulated with phorbol 12-myristate 13-acetate (PMA, 50 ng/mL, Sigma Aldrich, St. Louis, MO, USA), calcium ionophore (1 µg/mL, Sigma Aldrich), and lipopolysaccharide (LPS, 10 µg/mL, Sigma Aldrich), in the presence of brefeldin A (1 µg/mL) [44,45,46]. This stimulation strategy was selected to induce robust cytokine production across both T and B cell compartments, enabling comparative functional assessment independent of antigen specificity. Following incubation, erythrocytes were lysed using BD PharmLyse^TM^ Lysing Buffer (BD Biosciences). Cells were stained for surface markers (CD3, CD8, CD19, CXCR5), fixed and permeabilized using the Cytofix/Cytoperm kit (BD Biosciences), and subsequently stained for intracellular cytokines IFN-γ, IL-4, IL-10, and IL-21. Anti-CD4 antibody was included during intracellular staining to enhance identification of CD4^+^ cells following receptor internalization.

### 4.6. Flow Cytometry

Samples were acquired on an eight-color BD FACSCanto^TM^ II flow cytometer (BD Biosciences, San Jose, CA, USA) using BD FACSDiva software version 8.0.2. Compensation was established using unstained and single-stained controls, and final compensation matrices were generated during post-acquisition analysis in Infinicyt™ version 2.0 (Cytognos, Salamanca, Spain). Data analysis was performed using Infinicyt^TM^ and FlowJo™ version 10.6.2 (BD Biosciences). Initial gating was performed in Infinicyt^TM^ to identify lymphocytes, exclude doublets and artifacts, and define major lineages, after which files were exported to FlowJo^TM^ for detailed subset analysis.

### 4.7. Viral DNA Amplification by Real-Time qPCR

Plasma was separated from EDTA-anticoagulated blood by centrifugation at 900× *g* for 10 min. Viral DNA was extracted using the PureLink Genomic DNA Mini Kit (Invitrogen, Waltham, MA, USA). CMV DNA quantification was performed by real-time quantitative PCR using the forward primer 5′-CCC TCC GCG AAG CTC TTT-3′ and reverse primer 5′-CAG GTC CTC TTC CAC GTC AGA-3′. Reactions were prepared using LUNA Universal Probe qPCR Master Mix and the TaqMan probe 5′-TCG ACG TCA CCG TGG-3′. Amplification was carried out on a 7500 Fast Real-Time PCR System (Applied Biosystems, Waltham, MA, USA) with an initial denaturation step at 95°C for 10 min, followed by 40 cycles of 95 °C for 15 s and 60 °C for 1 min. All assays were performed using Sequence Detection Software version 1.3.

### 4.8. Statistical Analysis

Categorical variables are presented as absolute counts and percentages and were compared using the chi-square test or Fisher’s exact test, as appropriate. Continuous variables were assessed for normality by visual inspection of Q–Q plots, and where necessary, by the D’Agostino–Pearson test. Longitudinal changes in immune cell populations and soluble mediators were analyzed using mixed-effects models with participant as a random effect and trimester as a fixed effect, applying the Geisser–Greenhouse correction. Tukey’s test was used for multiple comparisons. Comparisons between CMV-seropositive and CMV-seronegative pairs were performed with an unpaired *t*-test with Welch’s correction. Comparisons between nonpregnant women and each pregnancy timepoint were performed using Brown–Forsythe and Welch ANOVA or ordinary one-way ANOVA with Dunnett’s correction. When parametric assumptions were not met, the Kruskal–Walli’s test with Dunn’s post hoc comparisons was applied.

To assess the combined effects of gestational stage and CMV serostatus, two-way mixed-effects ANOVA models were used, including interaction terms. Outliers were identified and excluded using the ROUT method (Q = 0.1%), as recommended by GraphPad Prism.

Statistical analyses and graphical representations mentioned above were generated using GraphPad Prism v10.4.0 for Windows (GraphPad Software, Boston, MA, USA).

Cytokine co-expression patterns were analyzed using a Boolean gating strategy to enumerate all possible combinations of IFN-γ, IL-4, IL-10, and IL-21 in CD4^+^ and CD8^+^ T cells and B cells. Net cytokine frequencies were calculated by subtracting the background values obtained under unstimulated conditions, and negative values were set to zero. A minimum frequency threshold of 0.2% was applied to exclude rare cytokine combinations.

Polyfunctionality analyses were performed using SPICE software version 6.1 [47]. Pie charts were generated to visualize the relative distribution of functional subsets, and statistical comparisons between cytokine-expression profiles were conducted using the SPICE permutation test (10,000 permutations). Individual cytokine combination frequencies were compared using the Wilcoxon rank-sum test.

All statistical tests were two-sided, and *p*-values < 0.05 were considered statistically significant.

## 5. Conclusions

This longitudinal study demonstrates that the combined influence of CMV seropositivity and pregnancy-associated immune remodeling shapes distinct maternal immune phenotypes, with pronounced effects on lymphocyte activation, differentiation, and functional capacity, particularly within the CD8^+^ T cell compartment. These immune signatures are a distinctive feature of CMV-seropositive pregnant women and are not fully recapitulated in the nonpregnant state. Importantly, CMV-seropositive individuals do not exhibit broad systemic inflammation during gestation, suggesting a predominantly cellular and functional mode of immune modulation.

Taken together, our findings highlight viral background as a key determinant of maternal immune variability during pregnancy and underscore the importance of considering CMV serostatus in immunological studies of gestation. Further investigation is warranted to define the clinical relevance of these immune alterations and their potential impact on maternal and fetal health.

## Figures and Tables

**Table 1:** Demographic, clinical, and laboratory characteristics of pregnant and nonpregnant women groups according to CMV serostatus.

Characteristics	PW CMV-IgG^+^ (*n* = 43)	PW CMV-IgG^−^ (*n* = 10)	NPW CMV-IgG^+^ (*n* = 25)	NPW CMV-IgG^−^ (*n* = 5)	*p*-Value
Age *, years	33.1 (4.0)	33.1 (3.8)	31.2 (4.7)	37 (7.3)	0.059 ^a^
≤33	24 (56)	6 (60)	17 (68)	2 (40)	0.612 ^b^
>33	19 (44)	4 (40)	8 (32)	3 (60)	
BMI *, kg/m^2^	25.2 (5.0)	23.6 (3.3)	23.7 (3.3)	21.8 (2.7)	0.262 ^a^
≥18.5 and <25	26 (61)	7 (70)	18 (72)	4 (80)	
≥25 and <30	10 (23)	2 (20)	5 (20)	1 (20)	0.972 ^b^
≥30	7 (16)	1 (10)	2 (8)	0 (0)	
Systolic pressure *, mmHg	118.0 (11.6)	117.4 (10.1)	118.7 (10.3)	112.4 (9.3)	0.701 ^a^
Diastolic pressure *, mmHg	72.4 (7.2)	69.5 (5.7)	72.4 (9.0)	70.1 (7.0)	0.719 ^a^
Parity, range	0–3	0–1	0–4	0–2	
Nulliparous	24 (55.8)	6 (60)	11 (44)	2 (40)	
Primiparous	11 (25.6)	4 (40)	7 (28)	0 (0)	0.197 ^b^
Multiparous	8 (18.6)	0 (0)	7 (28)	3 (60)	
Miscarriage history **, range	0–3	0–1	0–2	0–2	
<1	27 (62.8)	6 (60)	22 (88)	4 (80)	
≥1 and ≤2	15 (34.9)	4 (40)	3 (12)	1 (20)	0.226 ^b^
>2	1 (2.3)	0 (0)	0 (0)	0 (0)	
Gestational age at delivery, weeks	39.2 (1.1) ^1^	40.2 (0.8)	-	-	**0.008** ^d^
Type of delivery, *n* (%)			-	-	
Vaginal	25 (61)	7 (65)			0.597 ^c^
Cesarean	16 (39)	3 (35)			
Elective	2 (12.5)	0 (0)			>0.999 ^b^
Intrapartum	14 (87.5)	3 (100)			
Newborn’s birth weight, g	3187 (378) ^1^	3365 (336)	-	-	0.180 ^d^
Fetal sex, female, *n* (%)	17 (41.5)	5 (50)	-	-	0.489 ^c^
APGAR score, median [range]			-	-	
1 min	9.0 [6.0–10.0] ^1^	10 [9.0–10.0]			**0.008** ^e^
5 min	10 [9.0–10.0] ^1^	10 [10.0–10.0]			0.606 ^e^
Anti-CMV IgG, Log_10_(AU/mL)					
1st T	2.01 (0.27)				**<0.0001** ^a^ 0.403 ^f^
2nd T	1.98 (0.26) ^2^	-	2.37 (0.35)	-	
3rd T	1.98 (0.28) ^2^				

Data are listed as mean (standard deviation), range, or number *n* (%), otherwise indicated. * Data collected at study inclusion. ** Nonconsecutive episodes. ^1^ *n* = 41, ^2^ *n* = 18. ᵃ One-way ANOVA comparing mean values across all study groups. ᵇ Chi-square test comparing the distribution of categorical variables across groups. ᶜ Fisher’s exact test comparing the distribution of categorical variables across groups. ᵈ Unpaired *t*-test comparing normally distributed continuous variables between two independent groups. ᵉ Mann–Whitney U test comparing non-normally distributed continuous variables between two independent groups. ᶠ Mixed-effects model with Geisser–Greenhouse correction used to compare Anti-CMV IgG levels across trimesters within the same participants. APGAR normal range: 7–10. PW, pregnant women; NPW, nonpregnant women; BMI, body mass index; CMV, Cytomegalovirus; IgG^+^, seropositive; IgG^−^, seronegative. *p*-Values < 0.05 are highlighted in bold.

## Data Availability

The original contributions presented in the study are included in the article/Supplementary Material. Further inquiries can be directed to the corresponding author.

## Supplementary Materials

The following supporting information can be downloaded at: https://www.mdpi.com/article/10.3390/ijms27073257/s1.

## Abbreviations

The following abbreviations are used in this manuscript:

CMV	Cytomegalovirus
PW	Pregnant women
NPW	Nonpregnant women
Tfc	Follicular cytotoxic T cells
Tfh	Follicular helper T cells

## References

[1] Abu-Raya B., Michalski C., Sadarangani M., Lavoie P.M. (2020). Maternal Immunological Adaptation During Normal Pregnancy. Front. Immunol..

[2] Aghaeepour N., Ganio E.A., McIlwain D., Tsai A.S., Tingle M., Van Gassen S., Gaudilliere D.K., Baca Q., McNeil L., Okada R. (2017). An immune clock of human pregnancy. Sci. Immunol..

[3] Mor G., Aldo P., Alvero A.B. (2017). The unique immunological and microbial aspects of pregnancy. Nat. Rev. Immunol..

[4] Broekhuizen M., Hitzerd E., van den Bosch T.P.P., Dumas J., Verdijk R.M., van Rijn B.B., Danser A.H.J., van Eijck C.H.J., Reiss I.K.M., Mustafa D.A.M. (2021). The Placental Innate Immune System Is Altered in Early-Onset Preeclampsia, but Not in Late-Onset Preeclampsia. Front. Immunol..

[5] Deshmukh H., Way S.S. (2019). Immunological Basis for Recurrent Fetal Loss and Pregnancy Complications. Annu. Rev. Pathol. Mech. Dis..

[6] Zuhair M., Smit G.S.A., Wallis G., Jabbar F., Smith C., Devleesschauwer B., Griffiths P. (2019). Estimation of the worldwide seroprevalence of cytomegalovirus: A systematic review and meta-analysis. Rev. Med. Virol..

[7] Forte E., Zhang Z., Thorp E.B., Hummel M. (2020). Cytomegalovirus Latency and Reactivation: An Intricate Interplay With the Host Immune Response. Front. Cell. Infect. Microbiol..

[8] Liu X., Liu C., Zhang T. (2025). The Immunoregulatory Mechanisms of Human Cytomegalovirus from Primary Infection to Reactivation. Pathogens.

[9] Dollard S.C., Grosse S.D., Ross D.S. (2007). New estimates of the prevalence of neurological and sensory sequelae and mortality associated with congenital cytomegalovirus infection. Rev. Med. Virol..

[10] Kenneson A., Cannon M.J. (2007). Review and meta-analysis of the epidemiology of congenital cytomegalovirus (CMV) infection. Rev. Med. Virol..

[11] Rawlinson W.D., Boppana S.B., Fowler K.B., Kimberlin D.W., Lazzarotto T., Alain S., Daly K., Doutre S., Gibson L., Giles M.L. (2017). Congenital cytomegalovirus infection in pregnancy and the neonate: Consensus recommendations for prevention, diagnosis, and therapy. Lancet Infect. Dis..

[12] Pera A., Campos C., Corona A., Sanchez-Correa B., Tarazona R., Larbi A., Solana R. (2014). CMV latent infection improves CD8+ T response to SEB due to expansion of polyfunctional CD57+ cells in young individuals. PLoS ONE.

[13] Pera A., Vasudev A., Tan C., Kared H., Solana R., Larbi A. (2017). CMV induces expansion of highly polyfunctional CD4+ T cell subset coexpressing CD57 and CD154. J. Leukoc. Biol..

[14] Bayard C., Lepetitcorps H., Roux A., Larsen M., Fastenackels S., Salle V., Vieillard V., Marchant A., Stern M., Boddaert J. (2016). Coordinated expansion of both memory T cells and NK cells in response to CMV infection in humans. Eur. J. Immunol..

[15] Wirtz N., Schader S.I., Holtappels R., Simon C.O., Lemmermann N.A., Reddehase M.J., Podlech J. (2008). Polyclonal cytomegalovirus-specific antibodies not only prevent virus dissemination from the portal of entry but also inhibit focal virus spread within target tissues. Med. Microbiol. Immunol..

[16] Goldeck D., Oettinger L., Janssen N., Demuth I., Steinhagen-Thiessen E., Pawelec G. (2016). Cytomegalovirus Infection Minimally Affects the Frequencies of B-Cell Phenotypes in Peripheral Blood of Younger and Older Adults. Gerontology.

[17] Dauby N., Kummert C., Lecomte S., Liesnard C., Delforge M.L., Donner C., Marchant A. (2014). Primary human cytomegalovirus infection induces the expansion of virus-specific activated and atypical memory B cells. J. Infect. Dis..

[18] Lissauer D., Choudhary M., Pachnio A., Goodyear O., Moss P.A., Kilby M.D. (2011). Cytomegalovirus sero positivity dramatically alters the maternal CD8+ T cell repertoire and leads to the accumulation of highly differentiated memory cells during human pregnancy. Hum. Reprod..

[19] Taguchi A., Misumi F., Teraguchi S., Nagamatsu T., Sakakibara S., Otani T., Ichinose M., Priest D., Nakajima K., Nakamura J. (2025). Multifaceted profiling of virus-specific CD8 T cells reveals distinct immune signatures against cytomegalovirus infection states during pregnancy. iScience.

[20] Chidrawar S., Khan N., Wei W., McLarnon A., Smith N., Nayak L., Moss P. (2009). Cytomegalovirus-seropositivity has a profound influence on the magnitude of major lymphoid subsets within healthy individuals. Clin. Exp. Immunol..

[21] Leruez-Ville M., Foulon I., Pass R., Ville Y. (2020). Cytomegalovirus infection during pregnancy: State of the science. Am. J. Obstet. Gynecol..

[22] Papapavlou G., Hellberg S., Raffetseder J., Brynhildsen J., Gustafsson M., Jenmalm M.C., Ernerudh J. (2021). Differential effects of estradiol and progesterone on human T cell activation in vitro. Eur. J. Immunol..

[23] Schumacher A., Costa S.D., Zenclussen A.C. (2014). Endocrine factors modulating immune responses in pregnancy. Front. Immunol..

[24] Klenerman P., Oxenius A. (2016). T cell responses to cytomegalovirus. Nat. Rev. Immunol..

[25] Angelo-Dias M., Martins C.G., Mata M., Barata M., Chung A., Sarzedas S., Fernandes E., Appleton C., Lima J., Borrego L.M. (2025). Immunological reference intervals in pregnancy: Longitudinal analysis of adaptive lymphocyte subsets. Front. Immunol..

[26] Demery-Poulos C., Romero R., Xu Y., Arenas-Hernandez M., Miller D., Tao L., Galaz J., Farias-Jofre M., Bhatti G., Garcia-Flores V. (2022). Pregnancy imparts distinct systemic adaptive immune function. Am. J. Reprod. Immunol..

[27] Tarca A.L., Romero R., Xu Z., Gomez-Lopez N., Erez O., Hsu C.D., Hassan S.S., Carey V.J. (2019). Targeted expression profiling by RNA-Seq improves detection of cellular dynamics during pregnancy and identifies a role for T cells in term parturition. Sci. Rep..

[28] Gomez-Lopez N., Romero R., Galaz J., Bhatti G., Done B., Miller D., Ghita C., Motomura K., Farias-Jofre M., Jung E. (2022). Transcriptome changes in maternal peripheral blood during term parturition mimic perturbations preceding spontaneous preterm birthdagger. Biol. Reprod..

[29] Shah N.M., Herasimtschuk A.A., Boasso A., Benlahrech A., Fuchs D., Imami N., Johnson M.R. (2017). Changes in T Cell and Dendritic Cell Phenotype from Mid to Late Pregnancy Are Indicative of a Shift from Immune Tolerance to Immune Activation. Front. Immunol..

[30] Xie F., Hu Y., Magee L.A., Money D.M., Patrick D.M., Krajden M., Thomas E., von Dadelszen P., Toxemia Study G. (2010). An association between cytomegalovirus infection and pre-eclampsia: A case-control study and data synthesis. Acta Obstet. Gynecol. Scand..

[31] Balegamire S.J., Masse B., Audibert F., Lamarre V., Giguere Y., Forest J.C., Boucoiran I. (2024). Association Between Maternal Cytomegalovirus Seropositivity, Preterm Birth, and Preeclampsia in Two Cohorts From Quebec, Canada: A Mediation Analysis. Am. J. Reprod. Immunol..

[32] Geraili Z., Riahi S.M., Khani S., Rostami A., Bayani M., Hajian-Tilaki K., Nourollahpour Shiadeh M. (2018). Cytomegalovirus infection and risk of preeclampsia: A meta-analysis of observational studies. Caspian J. Intern. Med..

[33] Mocanu A.G., Stoian D.L., Daescu A.C., Motofelea A.C., Ciohat I.M., Navolan D.B., Vilibic-Cavlek T., Bogdanic M., Nemescu D., Tomescu L. (2024). The Impact of Latent Cytomegalovirus Infection on Spontaneous Abortion History and Pregnancy Outcomes in Romanian Pregnant Women. Microorganisms.

[34] Jarmund A.H., Giskeodegard G.F., Ryssdal M., Steinkjer B., Stokkeland L.M.T., Madssen T.S., Stafne S.N., Stridsklev S., Moholdt T., Heimstad R. (2021). Cytokine Patterns in Maternal Serum From First Trimester to Term and Beyond. Front. Immunol..

[35] Stokkeland L.M.T., Giskeodegard G.F., Stridsklev S., Ryan L., Steinkjer B., Tangeras L.H., Vanky E., Iversen A.C. (2019). Serum cytokine patterns in first half of pregnancy. Cytokine.

[36] Pang L., Wei Z., Li O., Huang R., Qin J., Chen H., Fan X., Chen Z.J. (2013). An increase in vascular endothelial growth factor (VEGF) and VEGF soluble receptor-1 (sFlt-1) are associated with early recurrent spontaneous abortion. PLoS ONE.

[37] Bolatai A., He Y., Wu N. (2022). Vascular endothelial growth factor and its receptors regulation in gestational diabetes mellitus and eclampsia. J. Transl. Med..

[38] van den Berg S.P.H., Pardieck I.N., Lanfermeijer J., Sauce D., Klenerman P., van Baarle D., Arens R. (2019). The hallmarks of CMV-specific CD8 T-cell differentiation. Med. Microbiol. Immunol..

[39] Hertoghs K.M., Moerland P.D., van Stijn A., Remmerswaal E.B., Yong S.L., van de Berg P.J., van Ham S.M., Baas F., ten Berge I.J., van Lier R.A. (2010). Molecular profiling of cytomegalovirus-induced human CD8+ T cell differentiation. J. Clin. Investig..

[40] Kim H.G., Sung J.H., Choi S.J., Oh S.Y., Roh C.R., Kim Y.J., Chang Y.S., Kim D.R., Moon I.J., Kang S.Y. (2025). Significance of placental pathology and CMV PCR in assessing clinical outcomes of congenital CMV infection. Placenta.

[41] Uenaka M., Morizane M., Tanimura K., Deguchi M., Kanzawa M., Itoh T., Yamada H. (2019). Histopathological analysis of placentas with congenital cytomegalovirus infection. Placenta.

[42] World Medical Association (2025). World Medical Association Declaration of Helsinki: Ethical Principles for Medical Research Involving Human Participants. JAMA.

[43] Sanz I., Wei C., Jenks S.A., Cashman K.S., Tipton C., Woodruff M.C., Hom J., Lee F.E. (2019). Challenges and Opportunities for Consistent Classification of Human B Cell and Plasma Cell Populations. Front. Immunol..

[44] Xu H., Liew L.N., Kuo I.C., Huang C.H., Goh D.L., Chua K.Y. (2008). The modulatory effects of lipopolysaccharide-stimulated B cells on differential T-cell polarization. Immunology.

[45] Mandala W., Harawa V., Munyenyembe A., Soko M., Longwe H. (2021). Optimization of stimulation and staining conditions for intracellular cytokine staining (ICS) for determination of cytokine-producing T cells and monocytes. Curr. Res. Immunol..

[46] Ai W., Li H., Song N., Li L., Chen H. (2013). Optimal method to stimulate cytokine production and its use in immunotoxicity assessment. Int. J. Environ. Res. Public Health.

[47] Roederer M., Nozzi J.L., Nason M.C. (2011). SPICE: Exploration and analysis of post-cytometric complex multivariate datasets. Cytom. Part A.

