# Supplementary material for: Prior Cytomegalovirus Infection Shapes Lymphocyte Activation and Function During Pregnancy
Source: Int J Mol Sci. 2026 Apr 3;27(7):3257. doi: 10.3390/ijms27073257 (PMC13073702; doi:10.3390/ijms27073257)
Supplement: Supplementary file 1 [file ijms-27-03257-s001.zip › Figure S1_new.pdf]

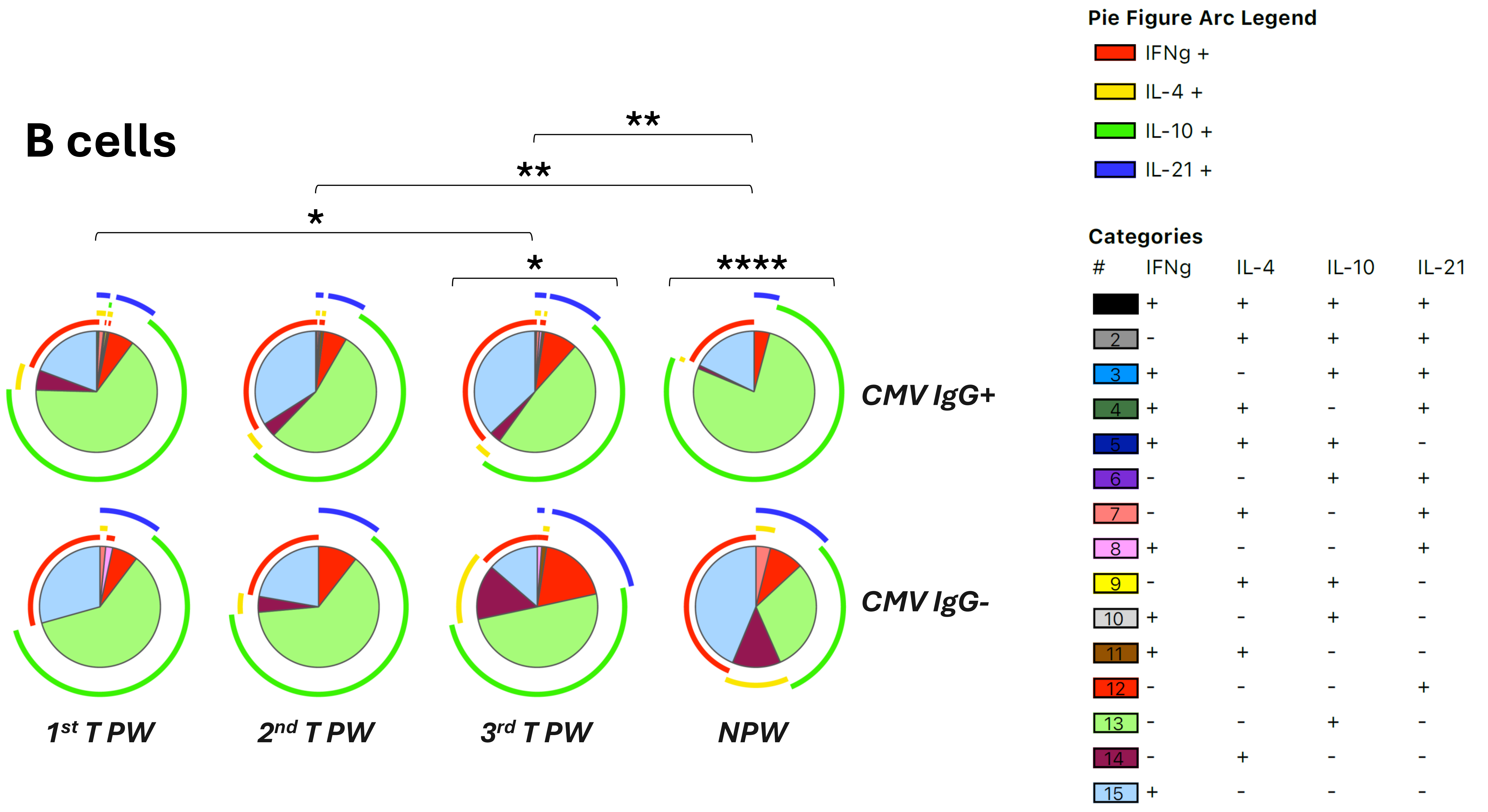


Supplementary Figure S1. Polyfunctionality of B cells after in vitro stimulation. Pie charts show the proportion of B cells producing different combinations of IFN-γ, IL-4, IL-10, and IL-21 following in-vitro stimulation with PMA, LPS, and ionomycin. Data are presented according to pregnancy stage (1st, 2nd, and 3rd trimester pregnant women, and nonpregnant women) and CMV serostatus (CMV-IgG+ and CMV-IgG-). Values shown in the pie charts represent mean frequencies. All frequencies were background-corrected by subtracting values obtained in the non-stimulated condition. Statistical differences between pie charts (indicated by brackers) were assessed using the SPICE permutation test (*p < 0.05, **p < 0.01, ****p < 0.0001). PW, pregnant women; NPW, nonpregnant women.
