# Supplementary material for: Prior Cytomegalovirus Infection Shapes Lymphocyte Activation and Function During Pregnancy
Source: Int J Mol Sci. 2026 Apr 3;27(7):3257. doi: 10.3390/ijms27073257 (PMC13073702; doi:10.3390/ijms27073257)
Supplement: Supplementary file 1 [file ijms-27-03257-s001.zip › Table S3.pdf]

Supplementary Table S3. Immunoassay analytical performance for serum cytokines and soluble mediators.

n=181	**LLOQ* ; ULOQ** (pg/m)**	**< LLOQ, n (%)**	**> ULOQ, n (%)**	**Non-detectable values, n (%)**
**IL-1α**	0.19 ; 226 - 302	75 (41.5)	0 (0)	20 (11.0)
**IL-1β**	0.26 ; 240 - 294	0 (0)	0 (0)	10 (5.5)
**IL-2**	2.97 ; 91 - 1109	102 (56.4)	0 (0)	56 (31.0)
**IL-4**	2.12 ; 337 - 363	118 (65.2)	0 (0)	2 (1.1)
**IL-6**	0.12 ; 656 - 732	0 (0)	0 (0)	0 (0)
**IL-8**	0.36 ; 818 - 949	0 (0)	3 (1.7)	0 (0)
**IL-10**	0.37 ; 602 - 784	34 (18.8)	0 (0)	0 (0)
**IFN-γ**	0.44 ; 246 - 433	126 (69.6)	0 (0)	12 (6.6)
**TNF-α**	0.59 ; 563 - 851	0 (0)	0 (0)	0 (0)
**VEGF**	3.81 ; 1353 - 1518	78 (43.1)	0 (0)	19 (19.5)
**MCP-1**	3.53 ; 593 - 843	0 (0)	0 (0)	0 (0)
**EGF**	1.04 ; 586 - 642	0 (0)	0 (0)	0 (0)
**VCAM-1*** **	7.3 ; 3310	0 (0)	0 (0)	0 (0)
**ICAM-1*** **	1.7 ; 940	0 (0)	0 (0)	0 (0)
**E-SEL*** **	1.6 ; 210	0 (0)	0 (0)	0 (0)
**P-SEL*** **	1.9 ; 1330	0 (0)	0 (0)	0 (0)
**L-SEL*** **	3.2 ; 4490	0 (0)	0 (0)	0 (0)

Lower (L-) and upper (U-) limit of quantification (LOQ). *This value is indicated by the manufacturer as functional sensitivity, which is defined as the lowest concentration with imprecision of ≤20% for 20 replicates. **ULOQ values are lot-dependent and are thus presented as interval. *** ng/ml
