# Supplementary figures and images for: Prior Cytomegalovirus Infection Shapes Lymphocyte Activation and Function During Pregnancy
Source: Int J Mol Sci. 2026 Apr 3;27(7):3257. doi: 10.3390/ijms27073257 (PMC13073702; doi:10.3390/ijms27073257)

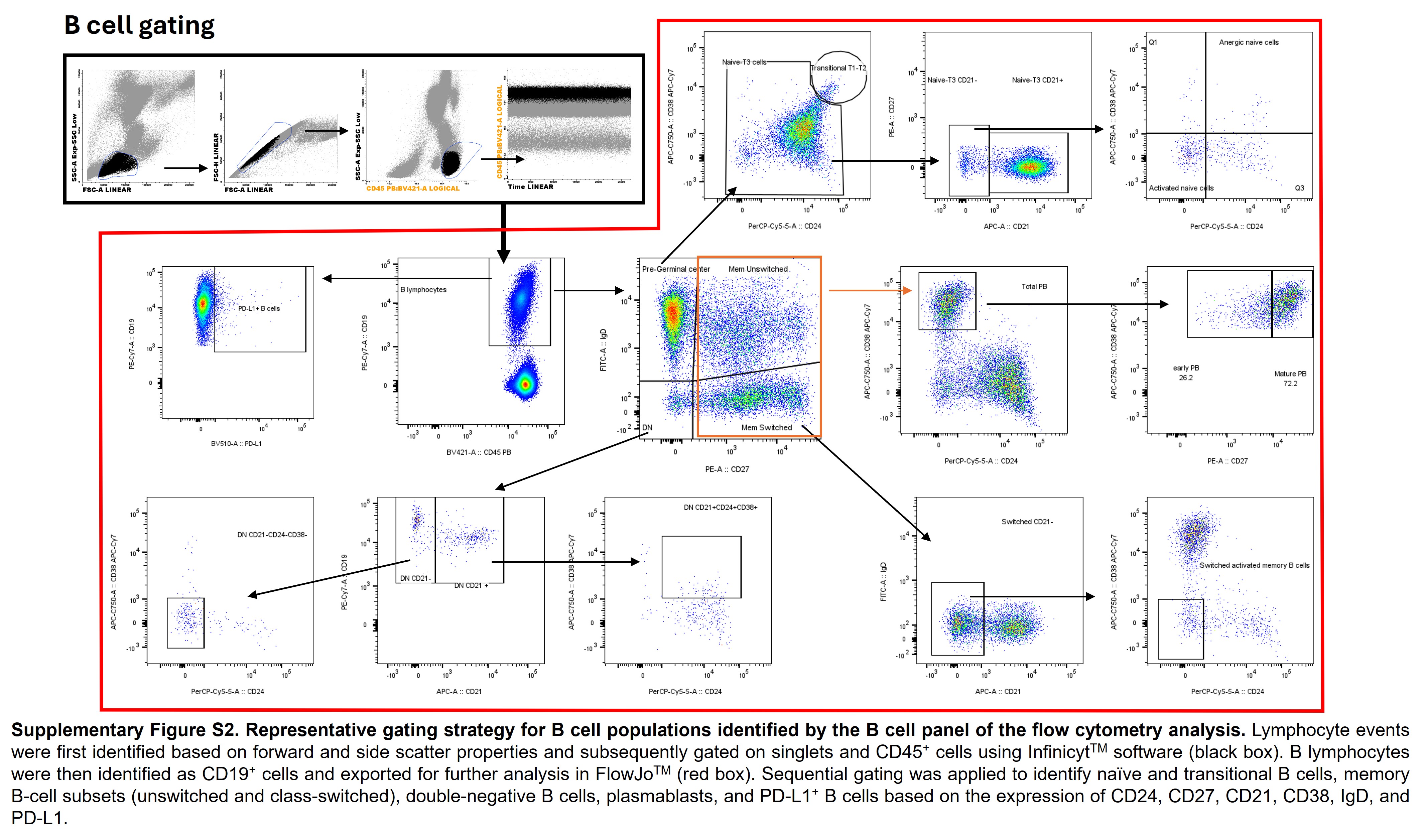

Supplement: Supplementary file 1 [file ijms-27-03257-s001.zip › Figure S2_NEW.jpg]

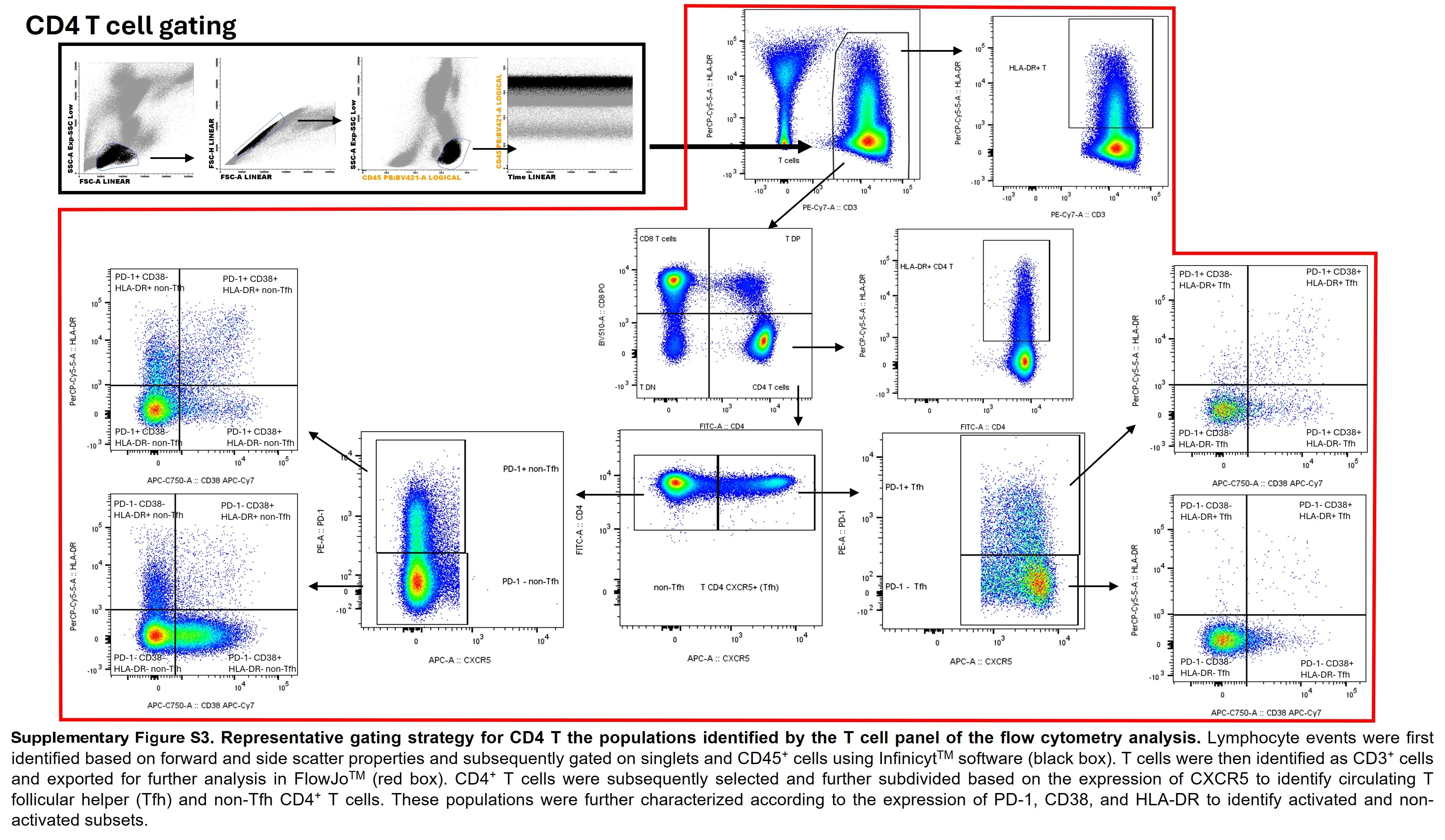

Supplement: Supplementary file 1 [file ijms-27-03257-s001.zip › Figure S3_NEW.jpg]

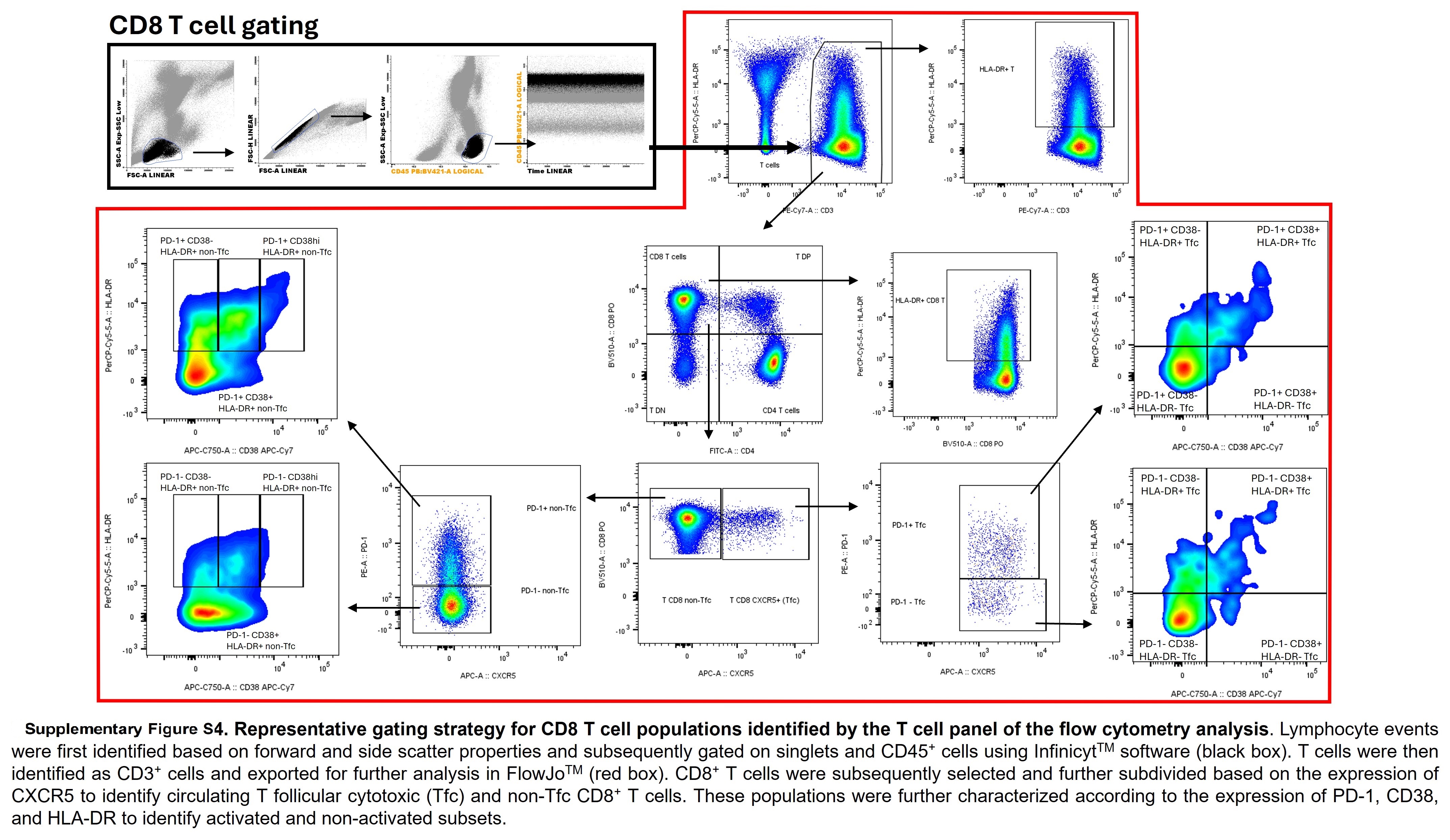

Supplement: Supplementary file 1 [file ijms-27-03257-s001.zip › Figure S4_NEW.jpg]

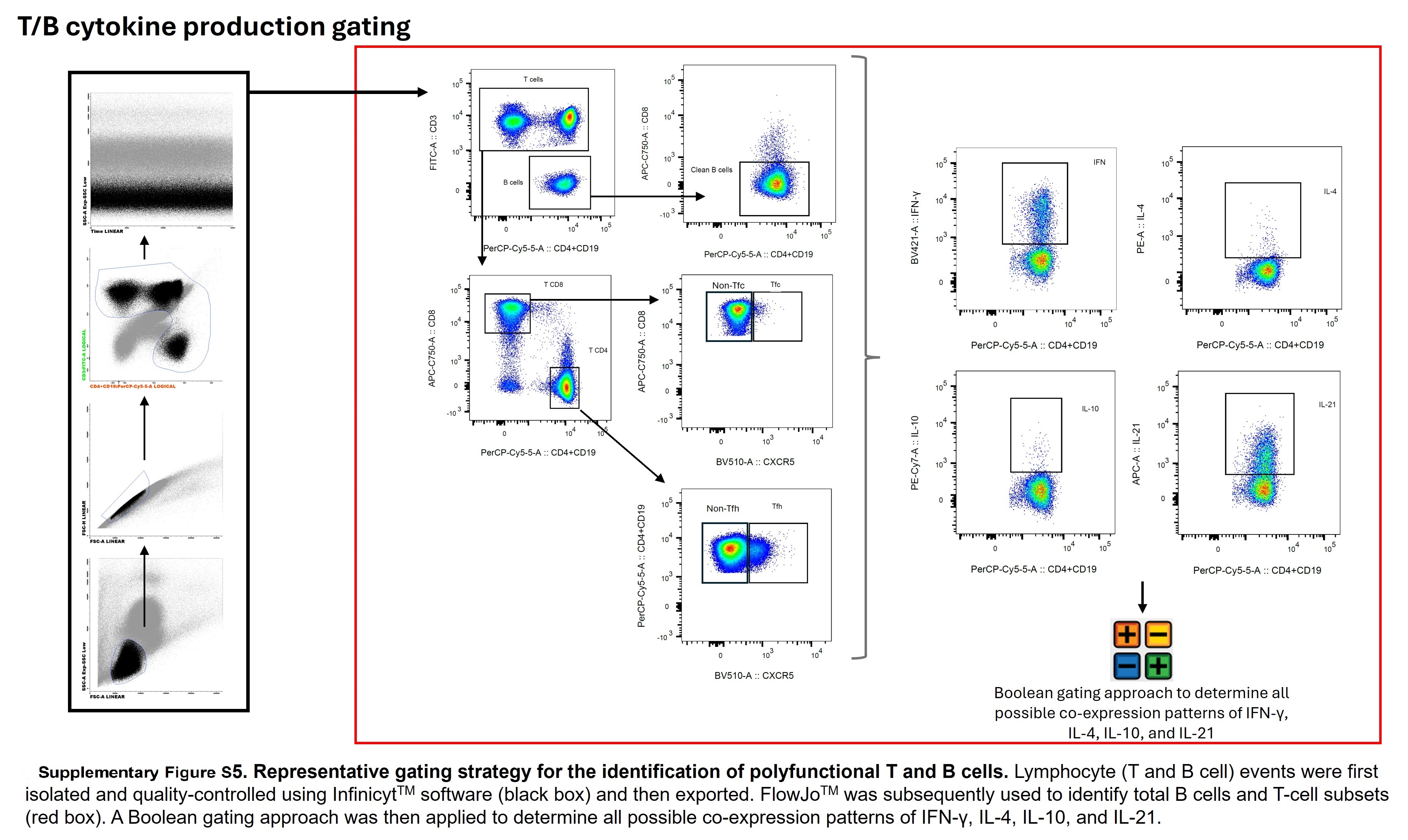

Supplement: Supplementary file 1 [file ijms-27-03257-s001.zip › Figure S5_NEW.jpg]
